# Naloxone for Severe Traumatic Brain Injury: A Meta-Analysis

**DOI:** 10.1371/journal.pone.0113093

**Published:** 2014-12-19

**Authors:** Hengzhu Zhang, Xiaodong Wang, Yuping Li, Renfei Du, Enxi Xu, Lun Dong, Xingdong Wang, Zhengcun Yan, Lujun Pang, Min Wei, Lei She

**Affiliations:** 1 Department of Neurosurgery, Clinical Medical College of Yang Zhou University, Yangzhou, Jiangsu Province, China; 2 Department of Neurosurgery, Chifeng Hospital, Chifeng, Neimenggu Province, China; 3 Department of Neurosurgery, The First People's Hospital of Zhenjiang, Zhenjiang, Jiangsu Province, China; Texas A&M University Health Science Center College of Medicine & Baylor Scott and White Health, United States of America

## Abstract

**Objective:**

The efficiency of naloxone for the management of secondary brain injury after severe traumatic brain injury (sTBI) remains undefined. The aim of this study is to evaluate the current evidence regarding the clinical efficiency and safety of naloxone as a treatment for sTBI in mainland China.

**Methodology/Principal Findings:**

A systematic search of the China Biology Medicine disc (CBM), China Science and Technology Journal Database (VIP), China National Knowledge Internet (CNKI), and Wan Fang Database was performed to identify randomized controlled trials (RCTs) of naloxone treatment for patients with sTBI in mainland China. The quality of the included trials was assessed, and the RevMan 5.1 software was employed to conduct this meta-analysis. Nineteen RCTs including 2332 patients were included in this study. The odds ratio (OR) showed statistically significant differences between the naloxone group and the control group (placebo) in terms of mortality at 18 months after treatment (OR, 0.51, 95%CI: 0.38–0.67; p<0.00001), prevalence of abnormal heart rates (OR, 0.30, 95%CI: 0.21–0.43; *p*<0.00001), abnormal breathing rate (OR, 0.25, 95%CI: 0.17–0.36; *p*<0.00001) at discharge, the level of intracranial pressure at discharge (OR, 2.00, 95%CI: 1.41–2.83; *p* = 0.0001), verbal or physical dysfunction rate (OR, 0.65, 95%CI: 0.43–0.98; *p* = 0.04), and severe disability rate (OR, 0.47, 95%CI: 0.30–0.73; *p* = 0.0001) at 18 months after the treatment. The mean difference (MD) showed statistically significant differences in awakening time at discharge (MD, −4.81, 95%CI: −5.49 to −4.12; *p*<0.00001), and GCS at 3 days (MD, 1.00, 95%CI: 0.70–1.30; *p*<0.00001) and 10 days (MD, 1.76, 95%CI: 1.55–1.97; *p*<0.00001) after treatment comparing naloxone with placebo group.

**Conclusions/Significance:**

This study indicated that applying naloxone in the early stage for sTBI patients might effectively reduce mortality, control intracranial pressure (ICP), and significantly improve the prognosis.

## Introduction

Severe traumatic brain injury (sTBI) occurs mainly in the young population and results in high morbidity and mortality. In China, more than 1/1000 people suffered from traumatic brain injury per year and the number has shown a substantial upward trend [1]. Although the treatment strategy for sTBI has been developed significantly in the past three decades, the mortality remains high (20% to 50%), which is commonly caused by brain swelling, cerebral infarction, delayed hematomas and cerebral hernia [2], [3]. Furthermore, the compression of brain blood vessels caused by diffuse brain swelling, cerebral contusion, and brain tissue hypoperfusion may lead to severe intracranial hypertension and even result in death. To prevent the secondary damage caused by uncontrollable intracranial hypertension, early decompressive craniotomy is a major strategy for sTBI and is widely used in China. A systematic review of retrospective case-control studies indicated that decompressive craniectomy could effectively reduce intracranial pressure. However, the results still needed to be evaluated by prospective randomized trials [4]. Some clinical trials suggested that after decompressive craniectomy, cerebral vascular perfusion pressure increased rapidly, which might aggravate cerebral edema and secondary brain injury [5]–[7]. Therefore, the current evidence suggested that adequate cerebral perfusion pressure was necessary to perform intracranial pressure control therapy. The American Trauma Foundations recommended that the ideal cerebral perfusion pressure for sTBI be between 50–70 mmHg [8].

Secondary brain injury plays a highly important role in the aggravation of sTBI. It has been found that secondary brain injury is caused mostly by an abnormal increase of endogenous β-opioid peptide in the traumatized brain tissue, which is closely related to patient prognosis [9]. As an opioid receptor antagonist, naloxone could exert a series of effects, including improving brain microcirculation, maintaining cerebral perfusion pressure, and preventing secondary brain damage. Some studies have indicated that the early application of high doses of naloxone could significantly reduce mortality in patients with acute brain injury, promoting good neurological function recovery and improving their prognosis [10], [11]. However, the efficacy and safety of the early usage of naloxone compared with placebo remains controversial.

Thus, we conduct this meta-analysis to assess the efficacy of naloxone versus placebo in treating patients with sTBI in mainland China in terms of overall mortality, the prevalence of abnormal vital signs, the level of intracranial pressure, awaken time, Glasgow Coma Scale (GCS), prevalence of verbal and physical dysfunction, the severe disability rate, and treatment-related complications.

## Materials and Methods

### Literature Search and Study Selection

A systematic literatures search including the China Biology Medicine disc (CBM, 1978–2013 Oct), China Science and Technology Journal Database (VIP, 1989–2013 Oct), China National Knowledge Internet (CNKI, 1994–2013 Oct), and Wan Fang Database (1997–2013 Oct) was performed (using the search terms “Naloxone”, “severe traumatic brain injury”, “TBI”, “brain injury”, “secondary brain injury”, and “randomized controlled trial”) to identify potentially relevant RCTs published in Chinese. The computer search mode of keywords combined with free words was adopted. All the search terms were performed the free words and Mesh terms searching. The search strategy was determined by two independent researchers. The search strategy of this meta-analysis is as follows:(“Naloxone”[All Fields] OR “Naloxone”[MeSH Terms]) AND (((((((“traumatic brain injury “[MeSH Terms] OR “traumatic brain injury”[All Fields]) OR “TBI”[MeSH Terms] OR “TBI”[All Fields]) OR “brain injury”[All Fields] OR “brain injury”[All Fields]) OR “secondary brain injury”[MeSH Terms] OR “secondary brain injury”[All Fields]) AND ((((“randomized controlled trial”[All Fields] OR “randomized controlled trials”[MeSH Terms]) OR “controlled clinical trial”[All Fields] OR “controlled clinical trial”[MeSH Terms]) OR “random allocation”[All Fields] OR “random allocation”[MeSH Terms]) OR “double-blind method”[All Fields] OR “double-blind method”[MeSH Terms]).

**Figure 1:**
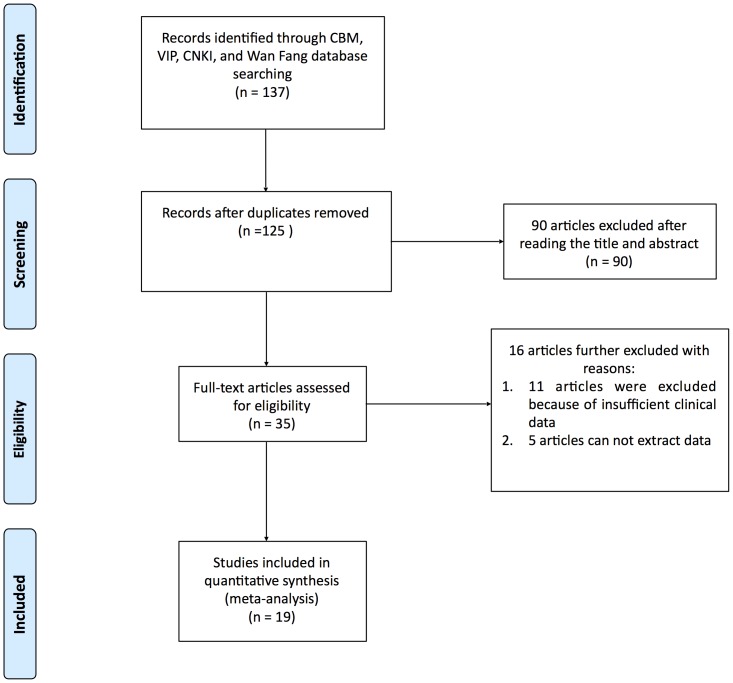
The PRISMA flow chart of the meta-analysis.

Two independent reviewers (XD.W and YP.L) assessed the literature based on the titles and abstracts to identify potentially relevant articles. Disagreements were resolved through discussion. Full versions of all relevant articles were obtained and inspected. The literature selection is presented in the PRISMA flow chart (Fig. 1) according to the PAISMA guidelines [12].

### Inclusion Criteria

The inclusion criteria were as follows: (1) randomized controlled trials (RCTs) published in Chinese; (2) naloxone treatment applied for sTBI and compared with placebo; (3) reports at least one of the main outcome measures of the study, including overall mortality, prevalence of abnormal heart rates, abnormal breathing, the level of intracranial pressure, awakening time, GCS score, prevalence of verbal and physical dysfunction, and the severe disability rate; (4) a minimum follow-up of 18 months; and (5) a sample size should larger than 20 patients in each group.

### Outcome Measures

The outcome measures included the following: (1) primary outcome measures: overall mortality, prevalence of abnormal heart rates (Pulse rate>110 bpm, Pulse rate <40 bpm), abnormal breathing (Respiratory rate>26 or <8 breaths/min Oxygen saturation <90% while on O_2_), and the level of intracranial pressure; and (2) secondary outcome measures: awakening time, GCS score, prevalence of verbal and physical dysfunction, and the severe disability rate.

### Literature screening and qualitative assessment

Two researchers (XD.W and YP.L) independently read the titles and abstracts of potential studies and requested the full texts to identify eligible research (meeting the inclusion criteria). The methodological quality was assessed according to the RCT evaluation criteria in Cochrane Reviewer's Handbook 5.0.0 [12], including the following: whether the randomization method was correct; whether allocation concealment was performed; whether a blinding method was conducted; and whether there were losses or exits from follow-up.

### Statistical Methods

The meta-analysis was performed using the statistical software RevMan5.1 (The Cochrane Collaboration). The odds ratio (OR) with 95% confidence interval (CI) was used as the effect indicator for the dichotomous variables, and the weighted mean difference (MD) was used for the measurement data. The heterogeneity assumption checked by the χ^2^-based *Q* test was used to test the clinical indicators. *P*>0.05 for the *Q* test indicates a lack of heterogeneity among the studies, and in such cases, the OR or MD estimate was calculated using the fixed-effects model (the Mantel-Haenszel method). Otherwise, the random-effects model (the DerSimonian and Laird method) was used. Sensitivity analysis was conducted to check the stability of results in each study, and the impact of different interventions was evaluated.

## Results

### Description of the Studies

Fig. 1 shows a flow chart of the study selection and inclusion process. The primary search yielded 137 potentially relevant articles (Fig. 1). Of these articles, 90 were excluded after reading the title and abstract. Then, the full text of the remaining 35 articles was read by 2 independent reviewers (YP.L and HZ.Z). Sixteen studies were further excluded because of insufficient clinical data (naloxone not compared with a placebo, 11 articles; data could not be extracted, 5 articles).

Based on the inclusion criteria, 19 RCTs including 2332 patients with sTBI were included in the meta-analysis [13]–[31]. The characteristics of the included studies are listed in Table 1. The sample size of the trials ranged from 40 to 512. All included studies were published in Chinese an described as RCT. There are 1122 sTBI patients in the naloxone group (48.11%) and 1200 patients in the placebo group. Five studies [14], [16], [18], [30], [31] were double blind, and the other studies did not report blinding.

**Table 1:** Characteristics of included studies.

Study	Gender F/M	Age	Year	Number of cases Naloxone/Control	Blind	Follow-up (m)	Quality
Yuchi Huang 13	51/18	31.3	2005	37/32	NR	24	B
Gang Yang 14	28/12	38	2001	22/18	Double-blind	18	A
Songtao Qi 15	97/49	28.4	2001	75/71	NR	24	B
Bing Chen 16	28/12	38	2005	20/20	Double-blind	22	A
Yimin Chen 17	NR	38.05	2005	62/69	NR	24	B
Zhixiong Huang 18	NR	NR	2001	20/20	Double-blind	24	A
Guoliang Guan 19	5432	36.95	2003	43/43	NR	20	B
Bangqing Yuan 20	46/30	28.4	2006	40/36	NR	30	B
Jie Cheng 21	132/104	44.55	2007	102/134	NR	24	B
Ling Ge 22	59/38	49	2010	38/39	NR	30	B
Youfen Xu 23	84/30	33.3	2000	56/58	NR	18	B
Jianjie Guo 24	72/28	31.24	2011	50/50	NR	18	B
Qiaoxian Fen 25	78/42	28	2006	62/58	NR	24	B
Baoming Wang 26	NR	NR	2006	41/38	NR	20	B
Junqing Li 27	82/26	35.3	2003	56/52	NR	24	B
Jiangguo Gao 28	78/42	28	2006	62/58	NR	22	B
Daqing Chen 29	58/24	37	2006	42/40	NR	18	B
Xiaoming Zen 30	52/24	33	2006	38/38	Double-blind	36	A
JinErLun Study Group 31	394/117	37.15	2011	256/255	Double-blind	24	A

NR: not report; Y: yes.

### Mortality (time frame)

**Figure 2:**
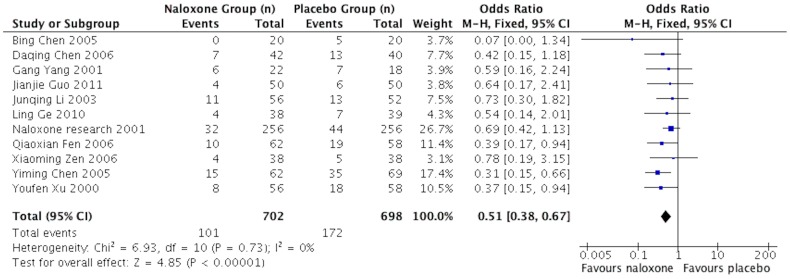
Meta-analysis of mortality of included studies.

Eleven studies [14], [16], [17], [22]–[25], [27], [29]–[31] reported the mortality of sTBI patients at 18 months follow-up end point. The test of heterogeneity showed no significant differences among the studies (I^2^ = 0); therefore, we applied the fixed-effects model. The mortality was 14.38% in the naloxone group compared with 24.64% in the placebo group. The pooled OR was 0.51 (95%CI: 0.38, 0.67; *p*<0.00001) (Fig. 2).

### Prevalence of abnormal heart rates and abnormal breathing

**Figure 3:**
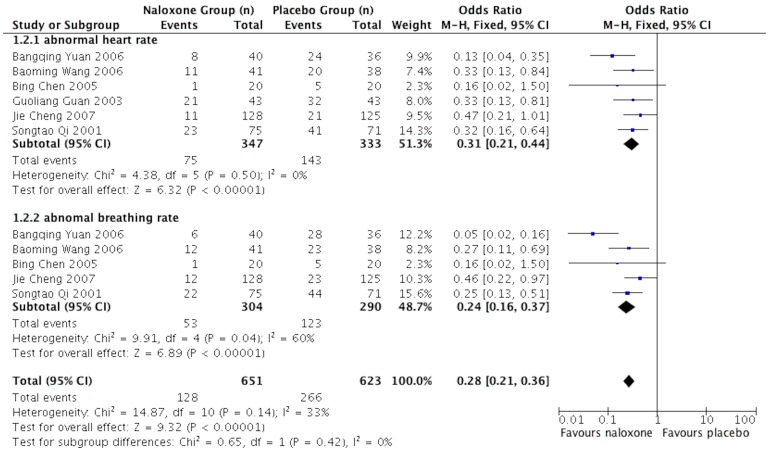
Meta-analysis of the prevalence of abnormal heart rates and breathing rate of naloxone for sTBI.

Six studies [15], [16], [19]–[21], [26] presented data on the prevalence of abnormal heart rates and abnormal breathing in sTBI patients who underwent naloxone or placebo treatment at discharge. The fixed-effects model was adopted because the heterogeneity analysis did not show a significant difference. The results showed that the prevalences of abnormal vital signs in the naloxone groups were significantly lower than in the placebo groups, and the pooled OR values for the prevalence of abnormal heart rates and the prevalence of abnormal breathing were 0.30 (95%CI: 0.21–0.43; *p*<0.00001) and 0.25 (95%CI: 0.17–0.36; *p*<0.00001), respectively. (Fig. 3)

### Intracranial pressure (time frame)

**Figure 4:**
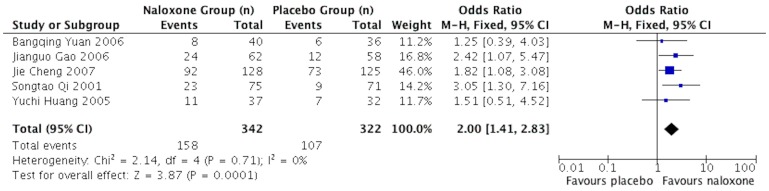
Meta-analysis of the level of intracerebral pressure of included studies.

Five studies [13], [15], [20], [21], [28] reported results on intracranial pressure at discharge. Naloxone more likely lowers the intracranial pressure of sTBI patients than placebo. The pooled OR was 2.00 (95%CI: 1.41–2.83; *p* = 0.0001) for low level ICP (<200 mmH2O), as shown in Fig. 4.

### Awakening time

**Figure 5:**
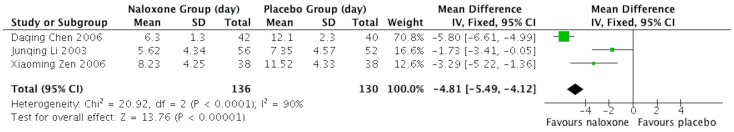
Meta-analysis of awakened time of sTBI patients after treatment.

Three studies [27], [28], [30] reported the awakening time of sTBI patients at discharge. The meta-analysis showed that naloxone promoted awakening better than placebo with statistical significance (MD, −4.81, 95%CI: −5.49 to −4.12; *p*<0.00001). (Fig. 5)

### The GCS scores

**Figure 6:**
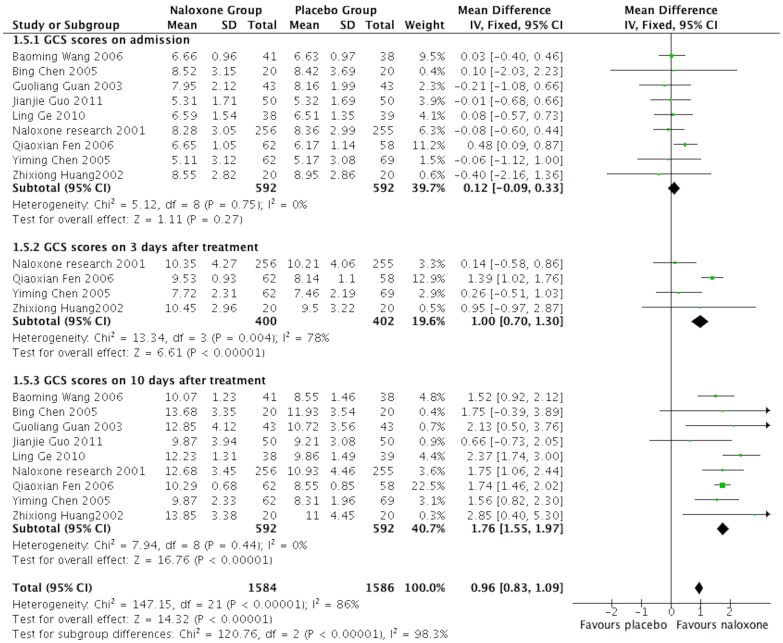
Meta-analysis of GCS in different time points.

Nine studies [16]–[19], [22], [24]–[26], [31] reported the GCS scores of patients on the day of admission and 3 days and 10 days after treatment. All the patients' GCS scores on the day of admission had a similar baseline without significant differences. The patients in the naloxone groups had significantly higher GCS scores than the placebo groups at 3 days (MD, 1.00, 95%CI: 0.70–1.30; *p*<0.00001) and 10 days (MD, 1.76, 95%CI: 1.55–1.97; *p*<0.00001) after treatment. (Fig. 6)

### Prevalence of verbal and physical dysfunction

**Figure 7:**
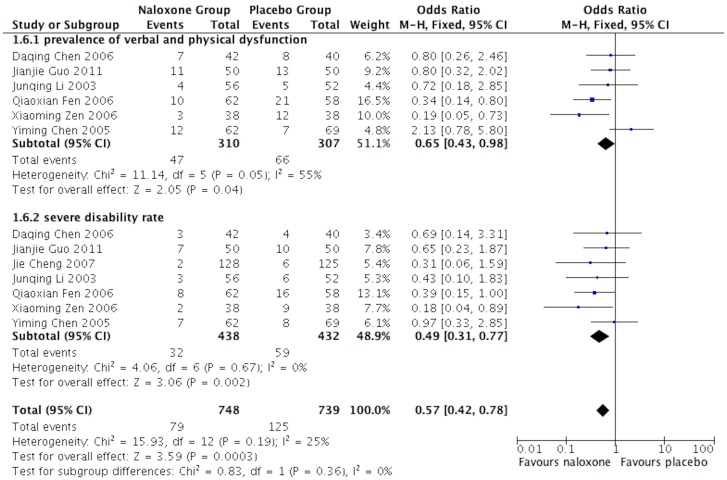
Meta-analysis of Prevalence of verbal and physical dysfunction in sTBI patients.

Seven studies [17], [21], [24], [25], [27], [29], [30] reported the prevalence of verbal and physical dysfunction (corresponding to a GOS of 4), and the severe disability rate (corresponding to a GOS of 2 and 3) of sTBI patients at 18 months after treatment. The test of heterogeneity showed no significant differences between the two groups, and therefore we applied the fixed-effects model. The results showed that naloxone could improve the patients' prognosis significantly compared with the placebo group. The pooled OR of the prevalence of verbal and physical dysfunction was 0.65 (95%CI: 0.43–0.98; *p* = 0.04), and the pooled OR for severe disability was 0.47 (95%CI: 0.30–0.73; *p* = 0.0001) (Fig.7).

### Qualitative Assessment and Publication Bias

**Figure 8:**
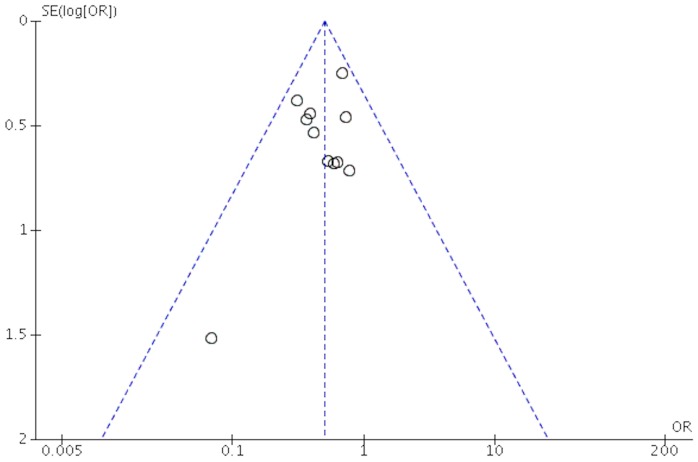
Funnel plot of included studies regarding morality.

The quality of the studies included in this meta-analysis is shown in Table 1. There were 5 studies conforming to grade A, while the other studies belonged to grade B according to the methodological quality assessment. It can be observed from the funnel plot that the publication bias was low regarding mortality (Fig. 8) and prevalence of abnormal heart rates and abnormal breathing (S1 Fig.), mediate regarding the level of intracranial pressure, GCS, and prevalence of verbal and physical dysfunction (S2, S4, and S5 Figs.), and high regarding awakening time (S3 Fig).

### Subgroup analysis of different doses and treatment durations

Dose and treatment duration may impact the mortality and prognosis of sTBI patients; therefore, a subgroup analysis was applied to determine the effect of dose and duration between the naloxone and placebo groups (Table 2). The subgroup analysis included two comparisons as follows: high-dose group (>0.3 mg/kg) versus low-dose group (<8 mg/d) and short duration (7–10 d) versus long duration (>14 d). The results showed no significant difference with *P* values in mortality of 0.58 and 0.99, GCS of 0.76 and 0.42, the preravence of verbal and physical dysfunction of 0.26 and 0.32, and severe disability of 0.97 and 0.68, respectively (test for subgroup differences).

**Table 2:** Subgroup analysis of different doses and treatment durations.

Outcome measures	variation		Included studies(n)	OR(95%CI)	P
Mortality					
	dose	<8 mg/d	5	0.46 [0.30, 0.70]	-
		>0.3 mg/kg	5	0.54 [0.37, 0.78]	0.58
	Treatment duration	7–10 days	5	0.52 [0.36, 0.76]	-
		14 days	3	0.43 [0.24, 0.78]	0.57
		>14 days	2	0.52 [0.27, 0.99]	0.99
GCS(10 days after treatment)					
	dose	<8 mg/d	3	1.83 [1.46, 2.21]	-
		>0.3 mg/kg	5	1.76 [1.51, 2.01]	0.76
	Treatment duration	7–10 days	6	1.66 [1.30, 2.03]	-
		>14 days	8	1.84 [1.59, 2.10]	0.42
verbal and physical dysfunction					
	dose	<8 mg/d	3	0.46 [0.24, 0.91]	-
		>0.3 mg/kg	2	0.80 [0.42, 1.51]	0.26
	Treatment duration	7–10 days	2	0.82 [0.40, 1.68]	-
		>14 days	3	0.51 [0.28, 0.93]	0.32
Severe disability					
	dose	<8 mg/d	4	0.46 [0.24, 0.89]	-
		>0.3 mg/kg	2	0.45 [0.20, 1.01]	0.97
	Treatment duration	7–10 days	2	0.53 [0.23, 1.23]	-
		>14 days	4	0.42 [0.22, 0.80]	0.68

P<0.05, show statistically significant differences.

### Sensitivity Analysis

We performed the sensitivity analysis on each study of this meta-analysis by deleting each individual data set to evaluate its influence on the pooled ORs. The results showed that no individual study significantly influence the pooled ORs.

## Discussion

TBI is a common severe disease that primarily occurs in patients below 45 years of age, with poor prognosis and a series of social problems. Because primary brain injury can cause several secondary complications, the mortality of sTBI patients is higher than for other traumatic injuries. Therefore, the prevention of secondary brain injury has become crucially important in clinical practice [32]. At present, the standard treatments of secondary brain injury in China are: 1) maintain airway patency, 2) early application of calcium antagonists, 3) mannitol, 4) neurotrophic drugs, 5) barbiturates, 6) high-dose corticosteroids, 7) Vitamin C, 8) hypothermia, and 9) surgery. The doctors apply the treatments according to the patient's condition. Xi's [15] study confirmed that patients in the naloxone group achieved reversed disturbance of consciousness and relieved respiratory depression more quickly than the placebo group (P<0.05). According to several studies focusing on the mechanism of secondary brain injury, a large quantity of endogenous endorphins have been found in TBI patients' cerebrospinal fluid, which might participate in the secondary brain injury procedure and be closely related to the prognosis of sTBI patients. Several studies have indicated that naloxone could effectively reduce the endogenous endorphin content and the reaction of inflammatory mediators, improve cerebral hypoxia, inhibit the generation of oxygen free radicals, resist lipid peroxidation, and protect the activity of the Na^+^-k^+^-ATP enzyme on the neuronal cell membrane [33]. Intracranial hypertension is another important cause of secondary brain injury. Therefore, it was necessary to monitor the patient's ICP level, which also assisted doctors in choosing the better treatment [34]. The results of this research showed that naloxone could effectively control intracranial pressure. However, the GCS scores of patients after naloxone treatments were much higher than for the placebo groups. This result indicated that early use of naloxone could help to protect brain neurons and promote neurological recovery.

The follow-up outcomes at 18 months after treatment showed that naloxone could reduce both mortality and the prevalence of verbal and physical dysfunction, improving the prognosis of patients. Yang's study [16] showed that naloxone could improve the conduction velocity of nerves and promote the recovery of neurological function.

The effective dose of naloxone remains controversial. According to a recent RCT, the usage of early high-dose naloxone achieved better efficacy than the low-dose group (*p*<0.0). Naloxone can penetrate the blood-brain barrier to result in a fast onset; however, its half-life is short. Some studies suggested that continuous administration of high-dose naloxone is essential to remain clinical efficacy [35]. However, our statistical analysis of subgroups found no significant difference between the high-dose and low-dose groups. To preclude the impact of the quality and sample size of the included studies, further studies are needed to determine the efficacy of different doses of naloxone for treating sTBI patients.

### Limitations

The included RCTs did not report the side effects of naloxone. In summary, this study revealed that naloxone could be recommended to treat sTBI patients, especially in the early stages. The limitations to this study were as follows: there were only 5 studies conforming to grade A, while the other studies belonged to grade B according to the methodological quality assessment; several RCTs needed improvements in the implementation of allocation concealment and the blinding method for evaluators and surveyors; the data on mortality and prognosis after 2 years have never been reported. In addition, all included clinical trials did not evaluate the mechanism action of naloxone, which might be of value to understand how naloxone worked in avoid secondary brain injury. These limitations might increase the possibility of publication bias and affect the final result of the meta-analysis. Therefore, further large multicenter RCTs are needed to confirm this conclusion.

## Conclusions

In summary, the results indicated that naloxone could effectively reduce the mortality and prevalence of abnormal vital signs, control the severe intracranial hypertension, shorten the awaken time, and improve the TBI patients' prognosis. In addition, naloxone might contribute to promoting the recovery of neurological function and significantly improve the prognosis of patients.

## Supporting Information

S1 Fig **Funnel plot of included studies regarding prevalence of abnormal heart rates and breathing rate.**
(TIFF)

S2 Fig **Funnel plot of included studies regarding the level of intracerebral pressure.**
(TIFF)

S3 Fig **Funnel plot of included studies regarding awakened time.**
(TIFF)

S4 Fig **Funnel plot of included studies regarding GCS in different time points.**
(TIFF)

S5 Fig **Funnel plot of included studies regarding Prevalence of verbal and physical dysfunction.**
(TIFF)

S1 Checklist **PRISMA checklist.**
(DOC)
