# Supplementary figures and images for: Naloxone for Severe Traumatic Brain Injury: A Meta-Analysis
Source: PLoS One. 2014 Dec 19;9(12):e113093. doi: 10.1371/journal.pone.0113093 (PMC4272270; doi:10.1371/journal.pone.0113093)

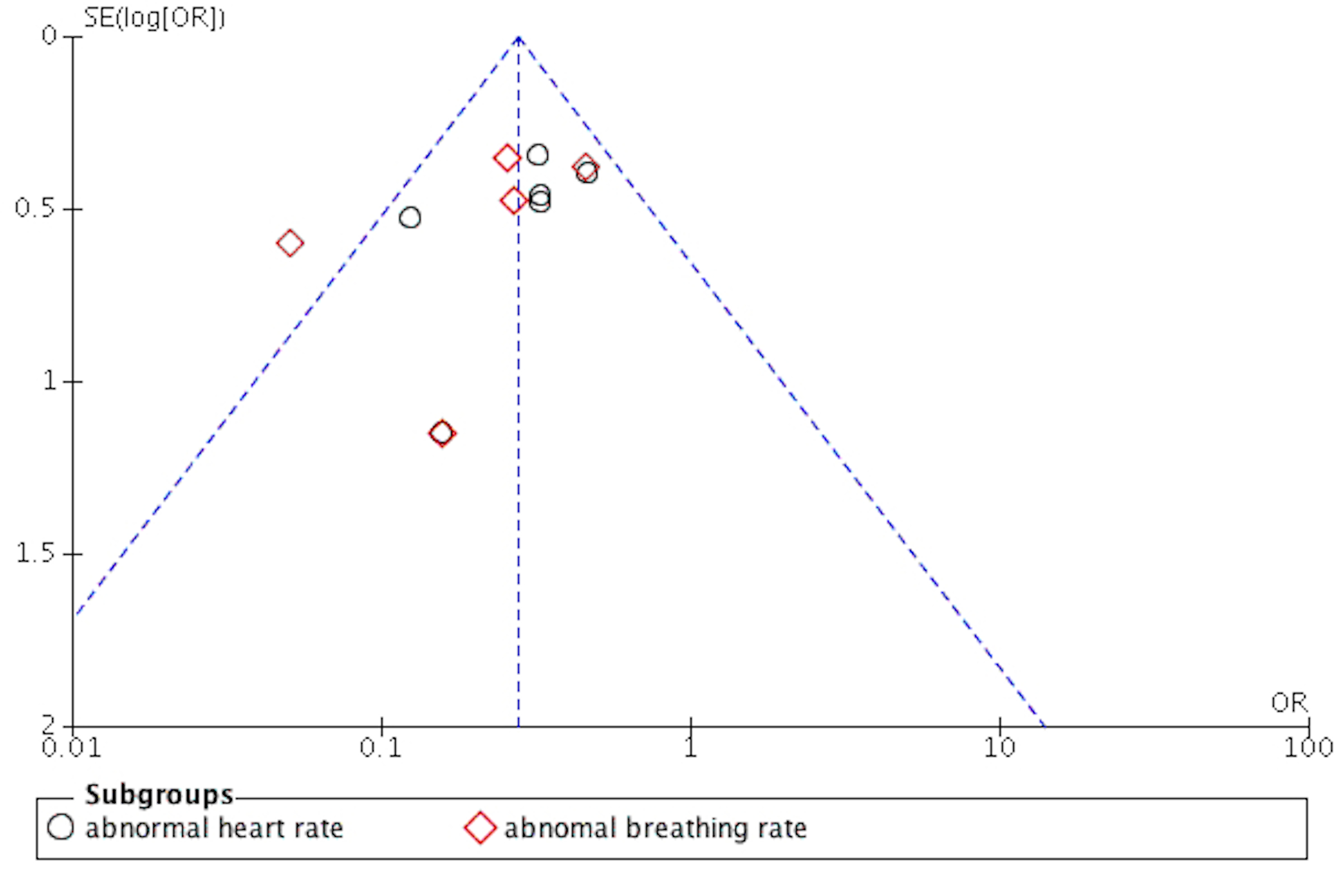

Supplement: S1 Fig — Funnel plot of included studies regarding prevalence of abnormal heart rates and breathing rate. (TIFF) [file pone.0113093.s001.tiff]

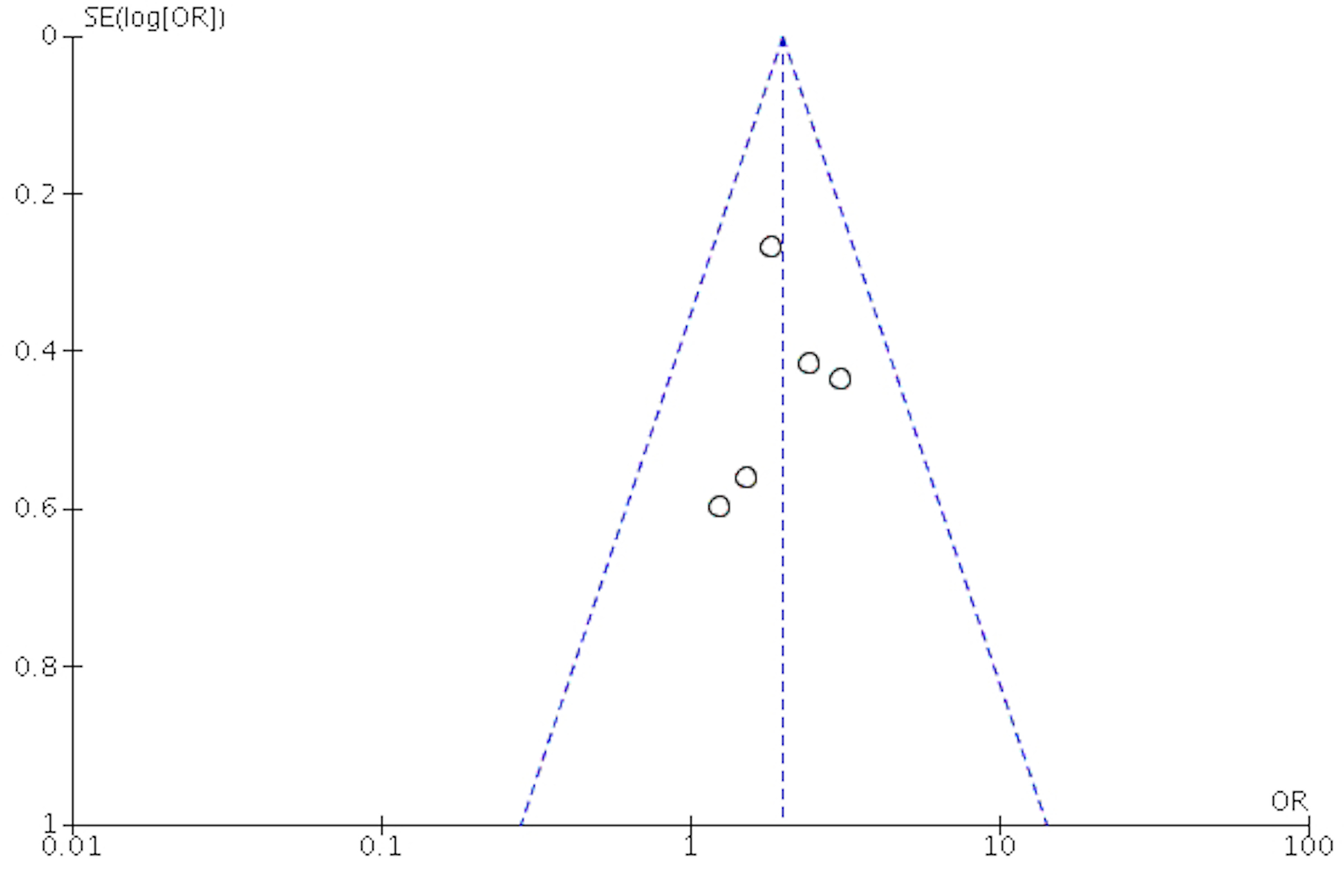

Supplement: S2 Fig — Funnel plot of included studies regarding the level of intracerebral pressure. (TIFF) [file pone.0113093.s002.tiff]

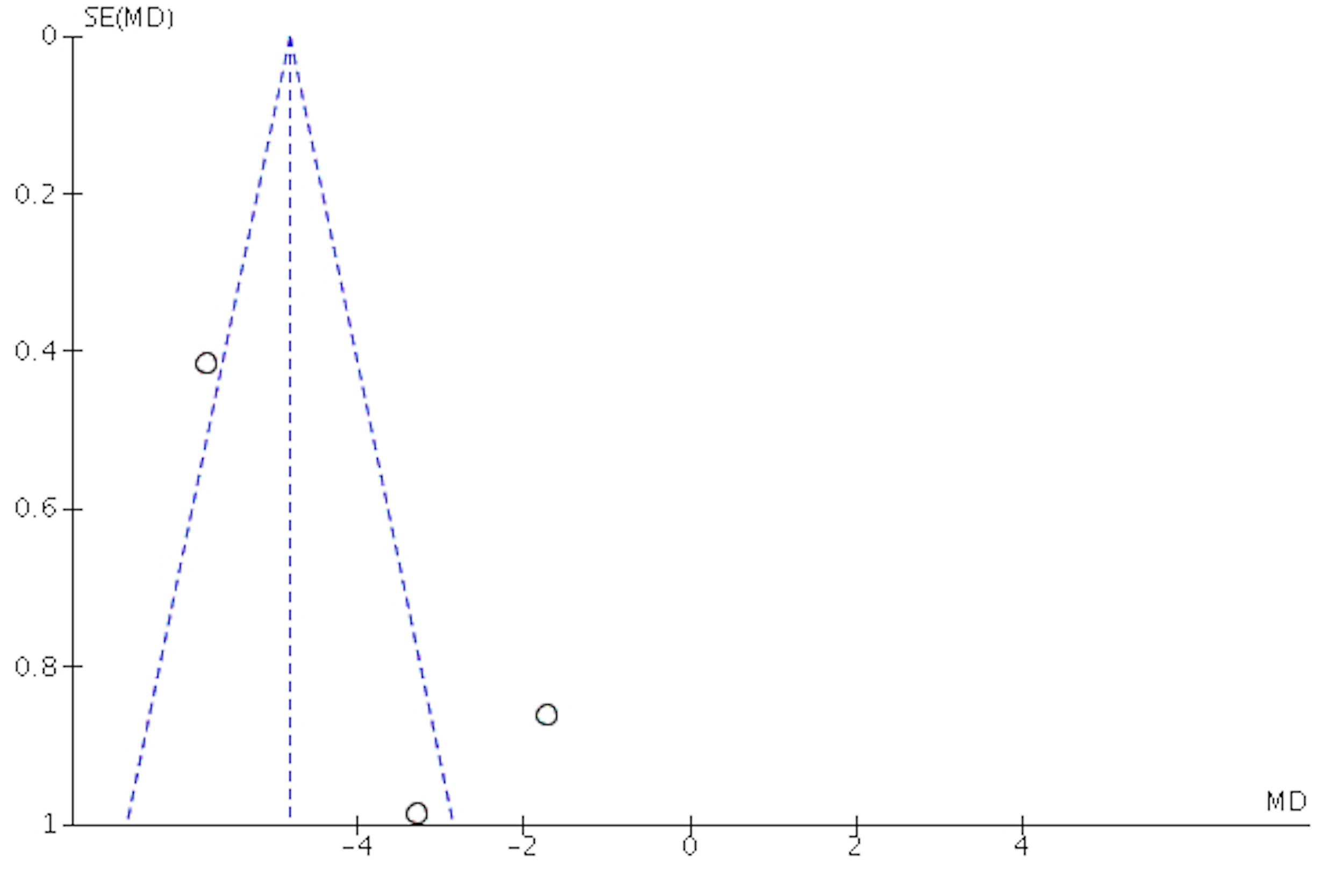

Supplement: S3 Fig — Funnel plot of included studies regarding awakened time. (TIFF) [file pone.0113093.s003.tiff]

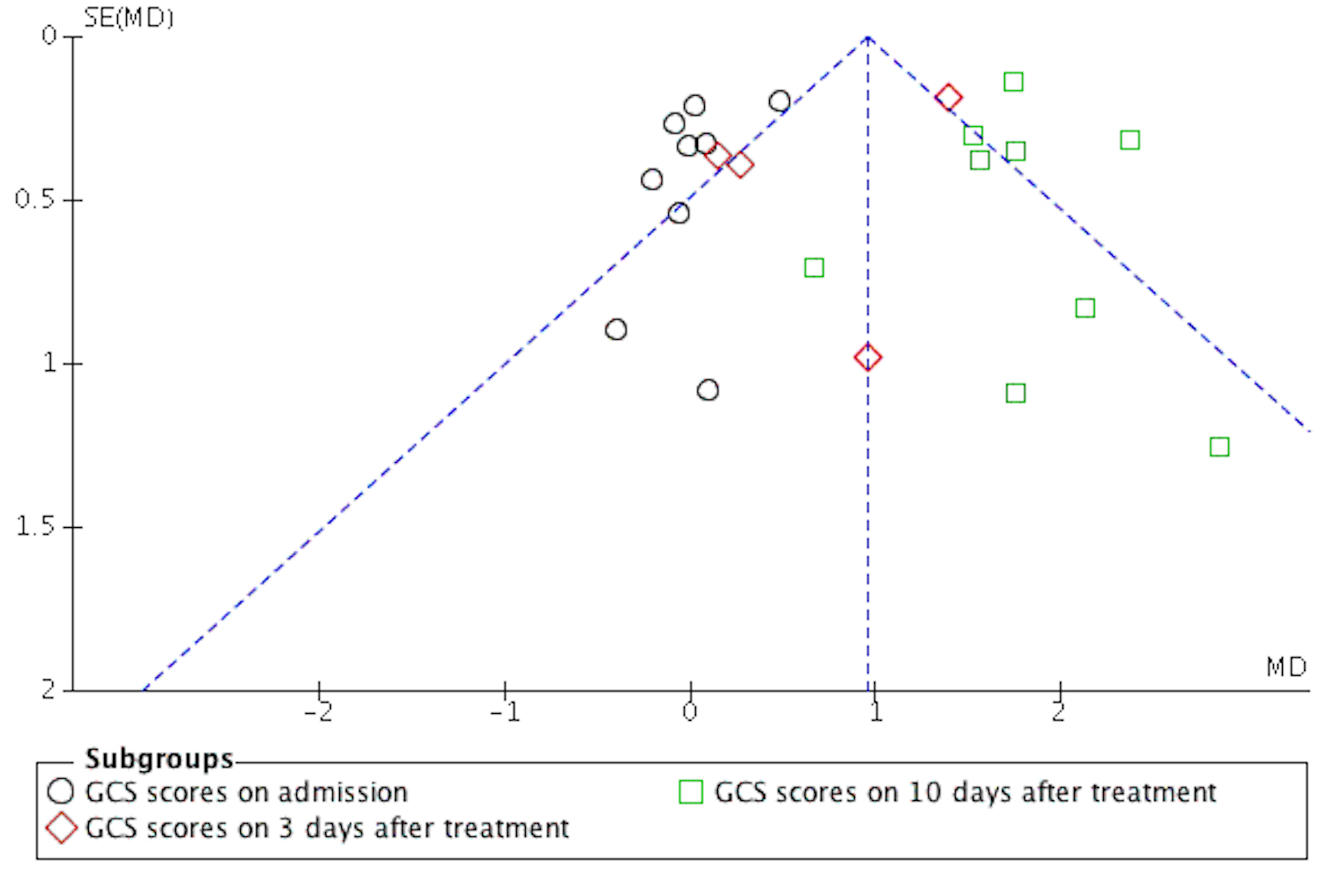

Supplement: S4 Fig — Funnel plot of included studies regarding GCS in different time points. (TIFF) [file pone.0113093.s004.tiff]

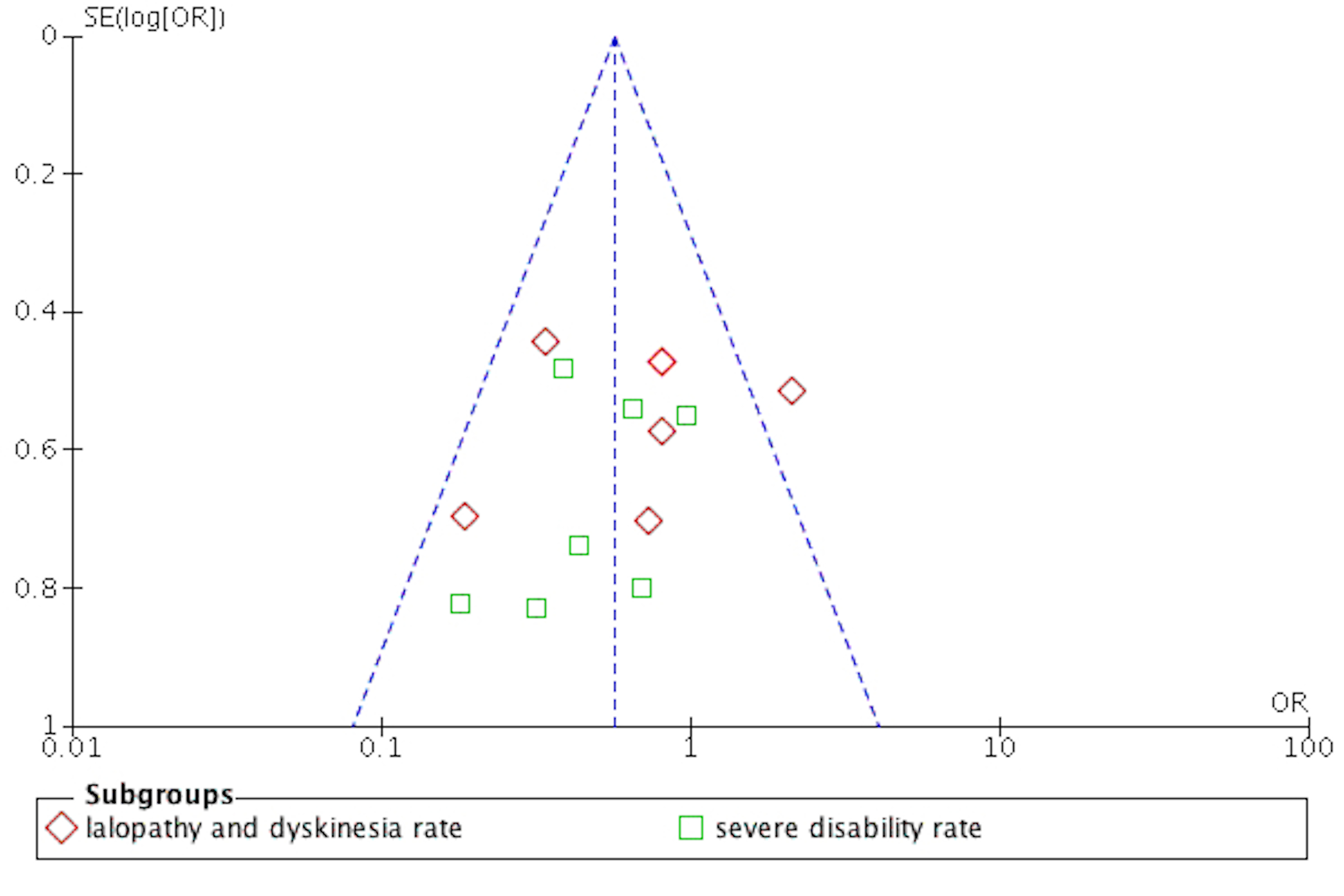

Supplement: S5 Fig — Funnel plot of included studies regarding Prevalence of verbal and physical dysfunction. (TIFF) [file pone.0113093.s005.tiff]
